# Cost, time savings and effectiveness of wearable devices for remote monitoring of patient rehabilitation after total knee arthroplasty: study protocol for a randomized controlled trial

**DOI:** 10.1186/s13018-023-03898-z

**Published:** 2023-06-27

**Authors:** Cheng Yang, Lei Shang, Shuxin Yao, Jianbing Ma, Chao Xu

**Affiliations:** 1 Department of Knee Joint Surgery, Honghui Hospital, Xi’an Jiaotong University, No. 555 E. Youyi Rd, Xi’an, Shaanxi China; 2 Department of Health Statistics, Faculty of Preventive Medicine, The Air Force Military Medical University, No.169 W. Changle Rd, Xi’an, Shaanxi China

**Keywords:** Total knee arthroplasty, Rehabilitation, Wearable devices, Telemedicine, Knee osteoarthritis

## Abstract

**Background:**

Total knee arthroplasty (TKA) is a surgical procedure primarily used to treat patients with end-stage knee osteoarthritis (KOA). Postoperative physical exercise is a critical part of the overall treatment of KOA and can bring significant benefits to the patients' recovery. Wearable devices can monitor patients' exercise data and upload it to the physician's workstation. This allows the rehabilitation physician to make timely adjustments based on the patients' movement feedback, and the surgeon can be informed of the patients' functional status. Overall, this study aims to evaluate the effectiveness of using wearable monitoring devices for rehabilitation exercise after TKA, with a focus on cost, time savings, and patient outcomes.

**Method/design:**

This is a single-center, single-blinded, parallel randomized controlled trial conducted at Xi'an Honghui Hospital, a regional orthopedic medical center. Eligible patients will be recruited to participate in the study, and baseline data collection and clinical assessments will be conducted at the time of admission. Using the principle of random allocation, recruited patients will be divided into either the experimental or control group. Both groups will undergo a standard, widely promoted rehabilitation program. The patients in the experimental group will wear equipment to detect and track mobility in the lower limbs. All patients will return to the outpatient clinic for follow-up assessments at 2 weeks, 12 weeks, and 24 weeks after discharge, where outcome indicators will be measured. The primary outcome will be the cost and time after discharge, while secondary outcomes will include the 6-min walk test (6MWT), range of motion (ROM), visual analog scale (VAS), American Knee Society Score (KSS), the Western Ontario and McMaster Universities Osteoarthritis Index (WOMAC).

**Discussion:**

We should encourage the adoption of novel, easy-to-use, supervised devices if they prove to be beneficial for patients in terms of cost, time, and effectiveness after TKA. This type of device is particularly important for people in remote rural areas, those with limited financial resources, and those who are reluctant to return to hospitals for follow-up care.

*Trial registration* Chinese Clinical Trial Registry ChiCTR2300068418. Registered on 17 February 2023.

**Supplementary Information:**

The online version contains supplementary material available at 10.1186/s13018-023-03898-z.

## Background

Osteoarthritis (OA) is a gradually advancing musculoskeletal disease that frequently affects the knee joint [1]. It is a debilitating condition and the primary indication for TKA [2]. TKA is a reliable surgical intervention for end-stage KOA [3, 4] and is recognized as a cost-effective treatment option [5]. The prevalence of TKA surgery has been increasing worldwide over the last 2 decades [6]. The rise in surgical procedures has imposed a substantial burden on healthcare budgets. Moreover, these surgeries are often accompanied by an extended rehabilitation period, significantly augmenting the costs of care [7].

Rehabilitation exercise after TKA is a critical component of a comprehensive KOA treatment plan and it can enhance patients' function, prognosis, and quality of life [8]. The rehabilitation program includes joint mobility exercises, such as range of motion, as well as exercises aimed at improving walking ability (posture, gait, and stability) and peripheral muscle strength [9]. The success of TKA relies on the effectiveness of postoperative rehabilitation and subsequent functional recovery [10]. Nowadays, home-based rehabilitation exercise, whether supervised or unsupervised, is considered a preferable choice for patients post-discharge due to its convenience [11–13]. Recent studies have revealed comparable outcomes between supervised physical exercise conducted at outpatient facilities and unsupervised exercise performed at home following TKA [14–18]. Systematic reviews and meta-analyses have further confirmed that supervised rehabilitation exercise does not provide any additional benefits [19, 20]. Hence, reducing costs and improving care efficiency are important objectives in rehabilitation programs [21]. Home-based rehabilitation offers a lower cost of care, aligning with this objective [22].

Wearable micro-sensing devices have become commonplace in the sports industry for real-time performance analysis of athletes [23, 24]. For instance, in the case of total hip arthroplasty and cardiovascular risk factors, accelerometry can be employed to monitor physical activity levels and estimate calories and energy expenditure by tracking movement patterns, including steps taken and distance traveled [25–27]. However, their potential use in healthcare has yet to be fully realized [28]. The number of studies on wearable motion sensors has grown in recent years, with many focused on knee replacements, providing additional information to clinical metrics [29]. This study aims to evaluate the cost, time savings and effectiveness of an innovative treatment approach using a new, simple monitoring device in conjunction with a mobile phone app to guide patient rehabilitation exercise. We hypothesize that patients in the experimental group would exhibit improved short-term functional outcomes and experience cost and time savings compared to patients in the control group.

## Methods/design

### Study design

**Fig. 1:**
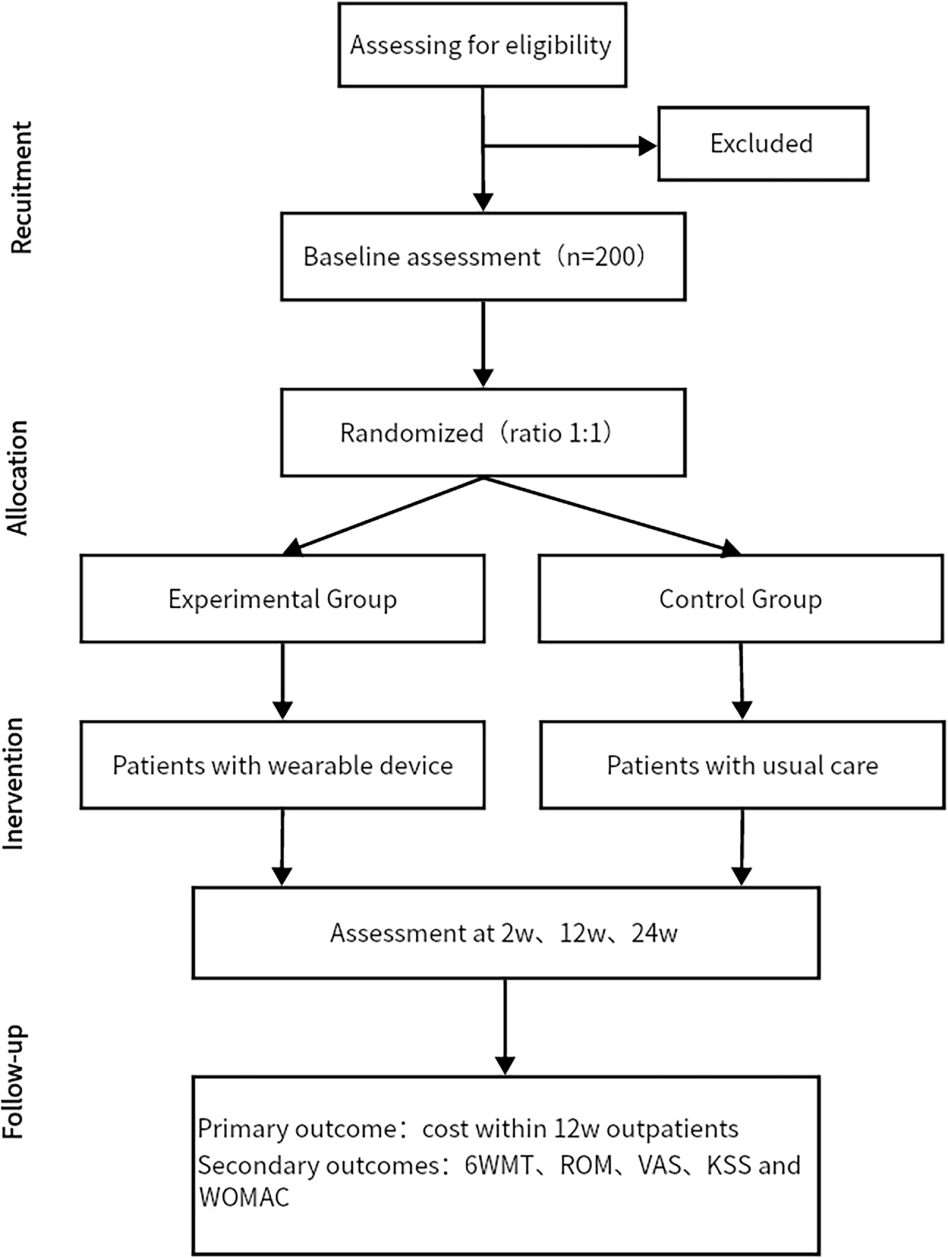
Proposed CONSORT diagram

This is a randomized controlled trial conducted at Xi'an Honghui Hospital. Patients are randomly assigned to one of two groups. The experimental group wears rehabilitation data acquisition devices and follows the APP guidance for exercise, while the control group follows the standard rehabilitation program. Both groups receive the same treatment regimen, rehabilitation exercise, and nursing services during hospitalization. Outcome assessments are conducted at 2w, 12w, and 24w after discharge, and the study flowchart is presented in Fig. 1. The study adheres to the Standard Protocol Items: Recommendations for Interventional Trials (SPIRIT) checklist, as provided in Additional file 1.

### Recruitment

Xi'an Honghui Hospital is a leading orthopedic center in China. The research team will provide patients with detailed information on the purpose, content, possible risks, and benefits of the trial, in adherence with the principle of informed and voluntary consent. The study will involve patients with end-stage KOA. Following completion of pre-operative preparations, TKA will be performed. The baseline registration and clinical evaluation conducted upon admission. During hospitalization, all patients receive the same treatment regimen and are discharged within 2–4 days after surgery.

### Inclusion criteria

The inclusion criteria for this study are as follows: (1) patients between 50 and 80 years old, (2) patients with end-stage knee osteoarthritis (KOA) who have been admitted to the hospital for TKA surgery, and (3) obtaining informed consent.

### Exclusion criteria

On the other hand, the exclusion criteria are: (1) patients who underwent surgical treatment of the lower limb within 6 months before or 3 months after this surgery, (2) patients with serious medical conditions that could affect exercise or outcome assessment, such as cerebral infarction, heart failure, or blood diseases, (3) patients with psychological disorders or neurological problems, (4) patients with cognitive deficits or language problems that would hinder their understanding of the study protocol, and (5) patients who refuse to participate.

### Exit criteria

The exit criteria for this study are as follows: (1) patients may withdraw from the study at any time without providing a reason, (2) patients experiencing adverse events such as wound dehiscence, infection, fracture, or pulmonary embolism, and (3) patients lost to follow-up. Any reasons for exit are recorded and reported to the ethics committee.

### Sample size

Some researchers recommend that the sample size for each endpoint should be calculated separately, and the largest sample size should be chosen as the overall trial's sample size [30]. The study is designed with two primary outcome measures: cost and time. By calculation, the sample size calculated based on cost is larger. Therefore, it is selected as the total sample size. The type I error α is set at 0.05 (2-tailed analysis), and the test efficacy 1 − *β* at 0.9. Based on the combined standard deviation of 3987 in previously published literature [31], we estimate that the difference in cost between the two groups within 12 weeks after surgery is approximately 2000 RMB. Using the formula $n = \frac{{2\left( {z_{\alpha } + z_{\beta } } \right)^{2} *\sigma^{2} }}{{\delta^{2} }}$, we determined that 80 patients are needed in each group. Accounting for a dropout and loss to follow-up rate of 20–25%, we need to recruit a total of 200 patients.

### Randomization and blinding

The 200 recruited patients are randomly assigned in a 1:1 ratio to either the experimental or control group using a computerized random number generator. Each patient is assigned a random number. Blinding is not possible due to the nature of the study, so neither patients nor physicians will be blinded to their group assignment. However, data analysts will not have access to any patient information other than their assigned number and will remain blinded during the statistical analysis of the collected data and presentation of results.

### Interventions

During their hospitalization, both the experimental and control groups receive the same treatment regimen, rehabilitation exercise, and nursing services. After undergoing TKA, patients in the experimental group are given wearable devices and are guided to perform rehabilitation training using an APP, while patients in the control group follow a handbook for their exercise program. The rehabilitation programs for both groups are consistent and were developed in accordance with the American Academy of Orthopaedic Surgeons (AAOS) Clinical Practice Guidelines (CGPs), the American Association of Hip and Knee Surgeons (AAHKS) guidelines, and other published literature [9, 32–34]. The rehabilitation exercise program can be found in Additional file 2. Following their discharge, both groups perform the home-based exercise program for 12 weeks. The patients in the experimental group will be required to return the devices to the research team at the 12th week. Following that, both groups of patients will no longer be required to adhere to the rehabilitation program's exercise regimen.

### Control group

Patients receive guidance from physiotherapists during their hospitalization, where they learn the necessary techniques for rehabilitation exercises. After being discharged, they continue with the rehabilitation exercise program using the handbook provided to improve their quality of life.

### Experimental group and equipment components

**Fig. 2:**
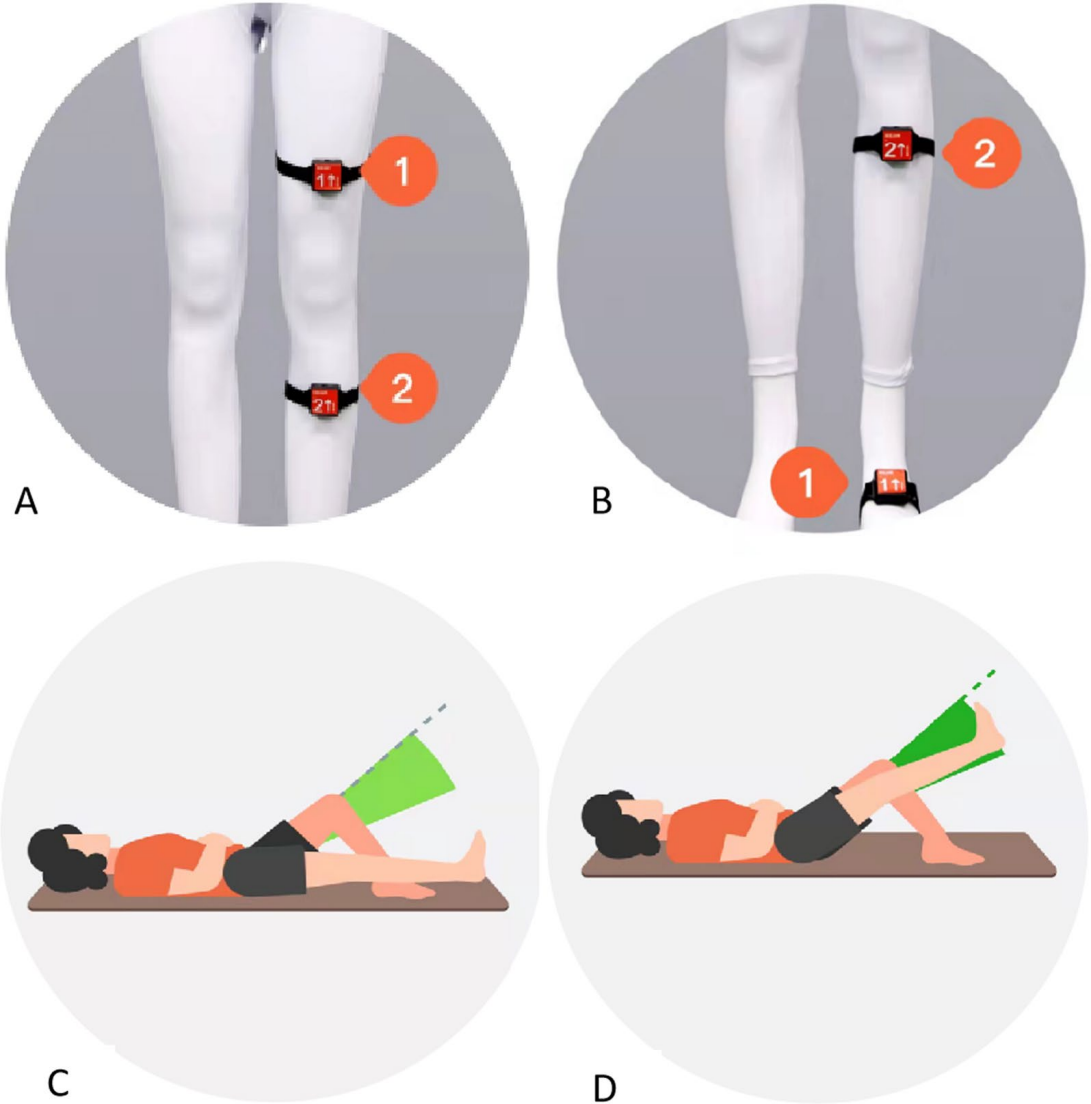
Diagram of wearable device usage. **A**, **B** The numbers and arrows are positioned on the front of the leg or foot and centered, as shown in the figure. **C**, **D** Straight leg raised to the green area is considered effective

The patients will require assistance from the doctors to download the APP and connect the devices. The devices consist of two small monitors, which are placed about 5 cm above and below the knee joint when exercising the knee joint, and about 5 cm below the knee and the dorsum of the foot when exercising the ankle joint (refer to Fig. 2). These devices can monitor the magnitude and completion of exercises and then upload the data to the physicians' workstation. Patients can watch instructional videos on the APP to learn how to wear and exercise with the devices and then start exercising actively. The APP provides prompts for exercise frequency and whether the exercise target is reached by voice. Patients can view their exercise progress on the APP, such as the completion rate and compliance rate. Additionally, they can upload their own functional and symptomatic information to the APP, such as lower limb swelling, ecchymosis, and wound healing, in the form of videos or pictures.

### Outcome measures

At the time of hospitalization, all recruited patients complete baseline registration which includes gender, age, BMI (kg/m^2^), education level, medical insurance, dwelling place, comorbidities, and medications. Clinical assessments such as knee extension and flexion, KSS, WOMAC, and the American Society of Anesthesiologists (ASA) physical status classification are also completed. At 2 weeks, 12 weeks, and 24 weeks after discharge, 6WMT, ROM, VAS, KSS, and WOMAC of patients are evaluated. The cost and time are calculated at 12 weeks after discharge.

### Primary outcome

The primary outcome measures of this study are the cost and time spent on medical care within 12 weeks after discharge. This includes costs for transportation, consultations, mobility aids, imaging examinations, and medications, as well as the time spent during the medical visit. All costs and time are recorded in a diary. Each entry in the diary must include the date, expenses, and time spent, along with the reasons. The diary is provided to the patients in both groups by the physiotherapists before discharge for free. The unit of cost is RMB, and the unit of time is hours.

### Secondary outcomes

Secondary outcomes consist of functional performance-based tests and rating scales. The 6MWT measures the distance a patient can walk on flat ground within 6 min. To assess ROM, the patient is required to lie flat on a bed or examination table, while the examiner measures the maximum angles of flexion and extension using a standard angle measuring instrument. VAS involves the use of a 10-cm-long line with anchor points at the ends, enabling the patient to self-assess and rate the intensity of pain. The 6MWT, ROM, and VAS can effectively reflect the knee function. In addition, the KSS and WOMAC provide a comprehensive evaluation of the patient's overall condition, including knee joint function, daily living ability, and quality of life.

### Data analysis

The study schedule is depicted in Table 1. Data of all patients are recorded in Excel format and imported into SPSS (version 26.0; Armonk, New York, USA) for statistical analysis. To minimize bias due to missing data, intention-to-treat analyses and multiple imputation methods are employed. Descriptive statistics of measurement data are presented as mean ± standard deviation, while counting data are expressed as rate and composition ratio. Continuous variables are subjected to Student's *T* test if they meet the normal distribution, and Wilcoxon rank sum test is used otherwise. Categorical variables are tested using the chi-square test or Fisher's exact test. Secondary analyses are adjusted for baseline characteristics using analysis of covariance (ANCOVA). This study involves conducting multiple hypothesis tests, which undoubtedly increases the probability of Type I errors significantly. To control for family-wise error, Bonferroni corrections are adopted, resulting in a lowered adjusted *p* value of 0.002.

**Table 1:** The patients’ schedule of trial enrollment, interventions, and assessment

Time point	Study period					
	Inpatient			Outpatient		
	Enrollment	Allocation	Pre-operation	2w	12w	24w
Enrollment						
Eligibility screen	×					
Informed consent	×					
Allocation		×				
Rehabilitation education			×			
Interventions						
Experimental group			×			
Control group			×			
Assessments						
Basal demographics and clinical data			×			
Cost					×	
Time					×	
6MWT						
ROM						
VAS						
KSS						
WOMAC						

“×” indicates the execution of the project according to the schedule

### Data management

The data monitoring committee, independent of the study group, is responsible for overseeing the preservation and safety of all subject data. Access to the data is restricted to researchers, and the test results will not be disclosed until the study is concluded.

### Ethics and dissemination

The study will adhere to the principles of the Declaration of Helsinki and Good Clinical Practice. The study protocol has been approved by the Ethics Committee of Xi'an Honghui Hospital (No.202301012) and registered in the Chinese Clinical Trial Registry (Registration Number: ChiCTR2300068418). The committee, which is independent of the research team, will oversee the trial's conduct. The research team will explain the study's purpose and methods in detail to potential participants or their authorized agents and obtain written informed consent from them. The team will also strictly safeguard the patients' data. Patients participating in the study have the right to discontinue their involvement at any time and without providing any reason, or the study may be discontinued if a clinician deems the patients unfit to continue due to other complications. Any adverse events will be reported to the ethics committee, and medical advice and assistance will be provided to the subjects. The trial data will be available to the subjects, and the results will be published in a journal.

## Discussion

The knee function and quality of life of patients generally improve significantly after TKA [35]. In 1997, the concept of enhanced recovery after surgery (ERAS) was introduced and quickly expanded to orthopedic applications, particularly TKA [36]. The goal of ERAS is to shorten hospital stays without increasing the risk of postoperative complications and readmission, reduce costs, and enhance patient satisfaction with surgical outcomes [37]. Given the COVID-19 pandemic, discharging patients as soon as possible after surgery to minimize exposure in healthcare settings is recommended [38].

Interest in telemedicine as a means of providing rehabilitation guidance after TKA is growing rapidly, fueled by the explosive growth of the Internet [22]. Digital health technology is an emerging telemedicine service that can remotely supervise patients for rehabilitation exercise [31, 39]. By using video or image interaction modes, patients can increase their interest and motivation for rehabilitation exercise [40]. Although supervised rehabilitation exercise has no additional functional benefits, telemedicine services are recommended to supervise patients in performing home-based rehabilitation exercise and improve their compliance [31, 40–42]. This study provides novel, simple, and supervised wearable devices that improve patients' sense of participation and provide timely feedback on their condition. This device will provide clinicians with a comprehensive understanding of the patient's physical health, enabling them to tailor treatment plans according to the patient's needs. Additionally, it can help overcome geographical and transportation barriers, particularly in remote rural and urban areas.

In conclusion, we anticipate that this device will bring benefits to patients. If successful, it has the potential to enhance medical services and warrant promotion, especially considering China's large and aging population.

### Limitations

Firstly, all participants were recruited from a single hospital located in the northwest region of China, which may limit generalizability to other populations. Secondly, there is a potential for response bias in this study, as the experimental group patients may alter their behavior when they are aware of being observed, showing greater compliance and less carelessness, and being more inclined to perform well. This potential for response bias may lead to exaggerate the differences in trial outcomes, and caution should be exercised when discussing the results.

### Trial status

Protocol version V1.1. The trial has not yet begun and is in the preparatory stages. Recruitment will begin on April 1, 2023, and a total of 200 patients will be enrolled, with completion expected before September 1, 2023.

## Supplementary Information

**Additional file 1.** SPIRIT Checklist.

**Additional file 2.** Rehabilitation program.

## Abbreviations

TKA: Total knee arthroplasty
OA: Osteoarthritis
APP: Application
6MWT: 6-Minute walk test
ROM: Range of motion
VAS: Visual analog scale
KSS: Knee Society Score
WOMAC: the Western Ontario and McMaster Universities Osteoarthritis Index
KOA: Knee osteoarthritis
SPIRIT: Standard Protocol Items: Recommendations for Interventional Trials
AAOS: American Academy of Orthopaedic Surgeons
CGPs: Clinical practice guidelines
AAHKS: American Association of Hip and Knee Surgeons
ASA: American Society of Anesthesiologists
ERAS: Enhanced recovery after surgery
