# Supplementary material for: Cost, time savings and effectiveness of wearable devices for remote monitoring of patient rehabilitation after total knee arthroplasty: study protocol for a randomized controlled trial
Source: J Orthop Surg Res. 2023 Jun 27;18:461. doi: 10.1186/s13018-023-03898-z (PMC10294457; doi:10.1186/s13018-023-03898-z)
Supplement: Supplementary file 2 — Additional file 2. Rehabilitation program. [file 13018_2023_3898_MOESM2_ESM.docx]

**Rehabilitation program**

| 2 weeks  Goal:1. Pain does not interfere with rest, 2. Knee flexion to 90°, 3. The back of the knee can be flat with the bed, 4. Be able to complete rehabilitation training by yourself | | |
| --- | --- | --- |
| Exercise | Description | Frequency |
| Ankle pumps | While lying supine with the leg extended, the patient moves the foot up and down while engaging the calf muscles | Repeat 10 times. Do 2 sessions per day |
| Straight leg raises | While lying supine with the leg extended, the patient raises the leg off the bed for a period of time | Hold 3-5 seconds. Repeat 10 times. Do 2 sessions per day |
| Calf raises | While standing with the aid of an assistive device, the patient lifts both heels off the ground and engages the calves; alternatively, the exercise is done on 1 leg | Hold 3-5 seconds. Repeat 10 times. Do 2 sessions per day |
| Bed-supported knee bends | While lying supine with the leg bent and keeping the bottom of the foot flat to the ground, the patient slides the heel toward the buttocks and holds the knee in the maximally bent position | Hold 30 seconds. Repeat 10 times. Do 2 sessions per day |
| Assisted knee bends | While lying supine with the operatively treated leg bent and keeping the bottom of the foot flat to the ground, the patient slides the leg toward the buttocks and holds it maximally bent but uses a towel wrapped around the leg to pull the leg closer to the body | Hold 30 seconds. Repeat 10 times. Do 2 sessions per day |
| Knee straightening | While lying supine with the leg extended but the heel of the foot held up by a small rolled-up towel, the patient attempts to drive the back of knee down toward the floor by engaging the quadriceps muscles | Hold 3-5 seconds. Repeat 10 times. Do 2 sessions per day |
| Hip bends resistance | While lying supine with the leg and hip bent, the patient pushes the knee with both hands against resistance. | Hold 3-5 seconds. Repeat 10 times. Do 2 sessions per day |
| Butt clamp | While lying supine with butt clamped, the patient feels the tightness and then relaxes | Hold 3-5 seconds. Repeat 10 times. Do 2 sessions per day |
| 2w-6w  Goal:1. Knee flexion to 100°,2. Gradually transition to walking with a single stick, 3. Basically return to normal gait, 4. Basic self-care at home | | |
| Ankle pumps | Mentioned above | - |
| Sitting knee straightening | While in a seated position with the legs hanging, the patient straightens the operatively treated leg and keeps the quadriceps taut | Hold 3-5 seconds. Repeat 10 times. Do 2 sessions per day |
| Standing knee bends | While standing with the aid of an assistive device, the patient lifts the operatively treated leg and bends the knee as much as possible | Hold 3-5 seconds. Repeat 10 times. Do 2 sessions per day |
| Center of gravity movement | While standing with one leg in front and the other leg in the back, the patient moves the center of gravity back and forth slowly, feeling the weight on lower limbs | Hold 3-5 seconds. Repeat 10 times. Do 2 sessions per day |
| Hip abduction | While in a lateral position with the operatively treated leg facing up and slightly stretched back, the patient lifts the leg and feels the force of the hip | Hold 3-5 seconds. Repeat 10 times. Do 2 sessions per day |
| Knee straightening | Mentioned above | - |
| Quadriceps sets | While lying supine with the leg extended, the patient tightens the knee to engage the quadriceps muscles; alternatively, the patient places a pillow under the knee, leaves the heel unsupported, and drives the knee down toward the floor through the pillow | Hold 3-5 seconds. Repeat 10 times. Do 2 sessions per day |
| Assisted knee bends | Mentioned above | - |
| Patellar mobility | While in a seated position with the leg straight, the patient pushes the patellar in all directions with hands | Repeat 10 times. Do 2 sessions per day |
| 6w-12w  Goal:1. Bend the knee to 110°, 2. Gradually transition to walking without a cane, 3. You can go to the supermarket and climb stair by yourself, 4. Can use toilet and stand up without armrest | | |
| Ankle pumps | Mentioned above | - |
| Standing knee bends | Mentioned above | - |
| Knee straightening | Mentioned above | - |
| Quadriceps weight bearing | While in a seated position with the legs hanging and a 2Kg sandbag tied around the ankle, the patient straightens the knee slowly | Hold 3-5 seconds. Repeat 10 times. Do 2 sessions per day |
| Center of gravity movement | Mentioned above | - |
| Hamstring pull | While in a seated position with the operatively treated leg straightening, the patient touches the toes with hands as much as possible to feel he tightness in the back of thigh | Hold 30 seconds. Repeat 10 times. Do 2 sessions per day |
| Standing hip abduction | While standing with a 3Kg sandbag tied the operatively treated leg, if unstable, the fixator can be held by hand, the patient abducts the hip | Hold 3-5 seconds. Repeat 10 times. Do 2 sessions per day |
| Standing hip bends | While standing with a 2Kg sandbag tied the ankle, the patient's leg is extended forward and the knee joint remains straight | Hold 3-5 seconds. Repeat 10 times. Do 2 sessions per day |
| Calf raises | Mentioned above | - |
| Hip abduction | Mentioned above | - |
| Assisted knee bends | Mentioned above | - |
| Sit-up | While in a seated position on the chair, the patient slowly rises and sits down with both hands holding the fixator | Hold 3-5 seconds. Repeat 10 times. Do 2 sessions per day |
| Patellar mobility | Mentioned above |  |
